# Appendiceal Carcinoids in Children–Prevalence, Treatment and Outcome in a Large Nationwide Pediatric Cohort

**DOI:** 10.3390/medicina59010080

**Published:** 2022-12-30

**Authors:** Johannes Wolfgang Duess, Ansgar Lange, Jan Zeidler, Jochen Blaser, Carmen Dingemann, Benno M. Ure, Martin Lacher, Jan-Hendrik Gosemann, Alejandro Daniel Hofmann

**Affiliations:** 1 Department of Pediatric Surgery, University of Leipzig, 04103 Leipzig, Germany; 2 Center for Health Economics Research Hannover (CHERH), Leibniz University Hannover, 30159 Hannover, Germany; 3 Techniker Health Insurance, Representative Officer of Lower Saxony, 30159 Hannover, Germany; 4 Department of Pediatric Surgery, Medical School Hannover, 30625 Hannover, Germany

**Keywords:** children, appendix, carcinoid, prevalence, surgical approach, follow-up

## Abstract

*Background and Objectives*: Appendiceal carcinoids are rare neuroendocrine tumors and mainly found incidentally during histopathological examination following appendectomy. This observational cohort study was performed to determine the prevalence, treatment modalities and outcomes in children diagnosed with an appendiceal carcinoid tumor. *Materials and Methods*: Data from the largest German statutory health insurance “Techniker Krankenkasse” were analyzed within an 8-year period: January 2010 to December 2012 and January 2016 to December 2020. Patient characteristics, surgical technique, type of surgical department, diagnostic management, and postoperative morbidity were analyzed. *Results*: Out of 40.499 patients following appendectomy, appendiceal carcinoids were found in 44 children, resulting in a prevalence of 0.11%. Mean age at appendectomy was 14.7 (±2.6) years. Laparoscopic approach was performed in 40 (91%) cases. Right-sided hemicolectomy was performed in 8 (18%) patients. Additional diagnostic work-up (CT and MRI) was recorded in 5 (11%) children. *Conclusions*: This large nationwide pediatric study shows that 1 in 1000 patients was found to have a neuroendocrine tumor of the appendix (prevalence 0.11%), emphasizing its low prevalence in the pediatric age group. The majority of patients were treated with appendectomy only. However, treatment modalities are still variable. Longer follow-up analyses are needed to evaluate published guidelines and recommendations to aim for a limited surgical approach.

## 1. Introduction

Neuroendocrine tumors of the appendix (appendiceal carcinoids) are the most frequent tumors of the gastrointestinal tract in children [1]. They are rarely associated with clinical symptoms and the vast majority of these tumors are found incidentally in children with appendicitis during histopathological examination of the resected appendix [2]. Following appendectomy, an incidence of 2 to 5 per 1000 cases has been stated [3,4,5]. However, data on the incidence in the current literature is misleading, and is merely presented as the frequency of carcinoid tumors in relation to the total number of appendectomies [6,7]. The reported occurrence of appendiceal carcinoids on the occasion of an appendectomy varies between 0.09% and 1.5% [5,7,8,9]. Appendiceal carcinoids are described to grow slowly and the overall prognosis is found to be excellent, using tumor size as an indicator for the assessment of malignancy [10]. Appendiceal tumors < 2 cm are considered to have no metastatic potential and lesions smaller than 1 cm can be treated by appendectomy only. In contrast, those exceeding 2 cm in diameter are often managed by right hemicolectomy (RHC). Nevertheless, controversy persists regarding the appropriate surgical management of children with a tumor size of 1–2 cm, and the role of a RHC in these patients [5,11,12].

Given the rare nature and the difficulty of preoperative diagnosis of appendiceal carcinoids, there is a paucity of pediatric cohorts in the literature [13]. Furthermore, available data of published studies are mainly based on reports from single centers and do not reflect a general approach towards the treatment of these neoplasms.

Using the database of a national major public health insurance, the aim of this study was to determine the prevalence, treatment modalities and outcomes in children and adolescents diagnosed with appendiceal carcinoid in a nationwide large pediatric cohort.

## 2. Materials and Methods

Claims data of the German public health care insurance company “Techniker Krankenkasse (TK)”, covering approximately 10% of the German population (approximately 9 million clients) were analyzed for this observational cohort study. The database proved anonymized information on patient characteristics, operative data, perioperative complications, readmissions and the treating surgical department. Patients with appendiceal carcinoid tumors were identified by the International classification of Diseases in its 10th version (ICD-10-GM). Surgical procedures were encoded based on the International Classification of Procedures in Medicine (ICPM). The study was approved by the Institutional Review Board of the Hannover Medical School (approval number 2647–2015).

### 2.1. Inclusion Criteria

All patients with the ICD-10 code “carcinoid tumor” and the ICPM code for “appendectomy” who were admitted from January 2010–December 2012 and from January 2016–December 2020 were analyzed. Due to regulations of the TK insurance it was not allowed to asses longer time periods. Therefore, this study was limited to two periods of 3 and 5 years. Only patients with complete datasets who had been continuous members of the TK during the study period were included.

### 2.2. Data Collection

Patient characteristics such as gender, age, surgical technique (laparoscopy/open), type of admitting department (pediatric surgery/general surgery/pediatrics/gynecology), diagnostic management and further surgical procedures (hemicolectomy, resection of the ileocecal valve) were analyzed at initial admission and within the first 180 postoperative days according to specific ICD10 and ICPM codes. Data are shown as mean (±SD) or number of cases (%).

## 3. Results

Out of a total number of 40.499 patients who underwent appendectomy between January 2010 and December 2012 (n = 10.014) and from January 2016 and December 2020 (n = 30.485), appendiceal carcinoid tumors were found in 44 patients, resulting in a prevalence rate of 0.11% (Table 1). Gender was recorded in 41 (93%) patients (n = 12; 29% males). In the remaining three (7%) children, gender was not indicated.

Three (7%) patients underwent abdominal CT scan prior to appendectomy. In one patient the tumor had already progressed with metastatic disease in the liver, lungs and axillary lymph nodes. Mean age at appendectomy was 14.7 (±2.6) years. In two (5%) patients age was not stated. Primary RHC was performed in a total of 2 (5%) children, including the patient with metastases found in the preoperative CT scan. An appendiceal carcinoid tumor was found in 10 (23%) patients younger than 13 years of age, whereas in patients aged 13 to 17 years and appendiceal lesion was diagnosed in 32 (73%) children. Mean length of initial hospital stay was 4.7 (±3.0) days. A laparoscopic approach was performed in 40 (91%) patients.

The majority of patients (n = 31; 76%) were operated by a general surgeon. Five (12%) children were admitted to a department of pediatrics, four (10%) to a pediatric surgical department and one 18-year old girl to a gynecological service. In three (7%) of the identified patients the admitting department was not stated.

Within the first 180 postoperative days, a total of 11 (25%) patients required further diagnostic or surgical treatment. Secondary RHC was performed in six (14%) patients. Five (11%) children underwent additional postoperative diagnostic work-up, including CT and MRI imaging, which was followed by a RHC in two patients after one month following initial appendectomy at a department of general surgery.

## 4. Discussion

### 4.1. Demographics and Prevalence

Although, neuroendocrine tumors of the gastrointestinal tract are rare, appendiceal carcinoids are the most common gastrointestinal epithelial tumors in children and adolescents with an overall reported prevalence up to 1.5% [5,8,9,14]. In our study, 44 children out of 40.499 cases were diagnosed with an appendiceal carcinoid tumor following appendectomy, resulting in a prevalence of 0.11%, which corresponds to the low rate of 0.169% reported by Doede et al. [7]. We only included cases with the ICD-10 code for “carcinoid tumor” and ICPM code for “appendectomy”, and therefore, incidental findings of appendiceal carcinoids found during laparotomies and laparoscopic procedures for a different pathology may have been disregarded and the exact frequency could be even higher. However, to the best of our knowledge, the amount of appendectomies analyzed in the present study from two different time periods is one of the largest cohorts available in a three-year and a five-year time period, respectively. We also believe that the acquisition of data from a health insurance company that covers 10% of a population does reflect a robust prevalence rate. In addition, incidence rates determined by several authors may not be accurate. Firstly, they are often presented as percentage of appendiceal carcinoids detected during appendectomies. Secondly, most neuroendocrine tumors of the appendix are found incidentally, therefore, the true incidence of these neoplasms is not known [6,7].

### 4.2. Diagnosis of Appendiceal Carcinoids

Neuroendocrine tumors of the appendix are typically identified during post-operative histopathological examination following appendectomy. Therefore, Coursey et al. conducted a study determining if carcinoid tumors of the appendix were identified on preoperative CT studies in adults, however could not detect any tumor prior to surgery. Carcinoids occurred in 1.1% of appendiceal specimens with less than 1.5 cm in size. Thus, the authors concluded that due to the small size these tumors were not identified prospectively [15]. We recorded 3 (7%) patients who underwent a preoperative CT scan in our study. In Germany, CT imaging in children and adolescents with acute abdominal pain is rarely performed to avoid the risk of ionizing radiation. Unfortunately, due to lack of clinical information in our database we could not determine the indications or results of those specific scans identified in our patient cohort retrospectively.

### 4.3. Age at Surgery and Surgical Approach

Mean age at appendectomy was 14.7 years in our study, which is similar to previous reports. Sommer et al., for example, have recently described a cohort and the management of pediatric patients with neuroendocrine tumors of the appendix in Switzerland. Out of 40 cases, the median age was 12.7 years and appendiceal carcinoids were more frequent in females [1]. In comparison to our cohort, we have also detected a female preponderance of 70%. However, due to the fact that we gathered our data from a nationwide database, this female predominance in our cohort is probably just a coincidence based on members of this insurance and therefore, does not reflect the “true” gender distribution among children with appendiceal carcinoids. Similar to a study by Gosemann et al., which analyzed the surgical approach towards appendectomy in Germany using data from the same health insurance, the majority of patients in this study were admitted to a general surgical department (76%) with a minimally invasive approach (91%) performed on most patients [16].

### 4.4. Malignancy of Neuroendocrine Tumors of the Appendix

Appendiceal carcinoids are considered potentially malignant tumors with rare local metastasis, including mesoappendiceal, periappendceal fat and regional lymphovascular invasion, but an overall excellent prognosis [8,17,18]. The role of serum markers (Chromogranin A (CgA), 5-hydroxyindoleacetic acid (5-HIAA)) and octreotide scintigraphy for the detection of local or distant metastasis is also still controversial as they lack sufficient sensitivity. In general, metastasis have been associated with larger tumor size and led to more extensive resection than simple removal of the appendix. In contrast, distant metastases have never been reported in the pediatric population so far [1,6,8,11,18]. In our analysis, one patient was identified with metastatic disease to the liver, lungs and axillary lymph nodes at the time of initial appendectomy and underwent RHC in the same procedure. Unfortunately, information on the outcome after our postoperative observation period could not be obtained.

### 4.5. Management of Neuroendocrine Tumors of the Appendix

Tumor size has been used to define surgical management, although the extent of resection beyond appendectomy remains controversial. Current consensus guidelines for adults published by several oncological societies and cancer networks across Europe and North America recommend performing RHC for a tumor size > 2 cm. Furthermore, RHC should be performed irrespective of tumor size in the presence of an invasion of the mesoappendix or incomplete R1 resection margin [19,20,21]. These recommendations have been claimed to be linked with adverse prognostic factors in adult studies [11]. However, guidelines in this age group may not translate to the pediatric population and due to the scarcity of appendiceal carcinoid tumors in children, specific pediatric guidelines have been difficult to establish with variable treatment options of these tumor entities [5,11]. Nevertheless, there are numerous recommendations for additional surgery and follow-up procedures in children mainly based on findings from retrospective analyses, which are summarized in Table 2. While some advocate extensive surgery, such as ileocecal pole resection or RHC for a tumor size > 1.5 cm or in the presence of regional metastases, others favor a less aggressive approach due to the benign clinical course and excellent prognosis (Table 2). The duration for postoperative surveillance is quite variable as well and the use of imaging procedures or biomarkers during follow-ups (Table 2). In 2013, the German Society for Pediatric Oncology and Hematology (GPOH) published data of children with appendiceal neuroendocrine tumors since 1997 (GPOH-MET trial). The objective of this study was to critically evaluate the therapeutic recommendations for appendiceal neuroendocrine tumors in children. The GPOH has, therefore, recommended secondary RHC in completely removed appendiceal neuroendocrine tumors of >1.5 cm in size. For incompletely removed tumors ≤ 1.5 cm a local follow-up resection with lymph node sampling was advised [22] (Table 2). Nevertheless, appendectomy only might be an adequate treatment for appendiceal tumors in general as recently stated by a Parikh et al. The authors conducted a nationwide study based on the database of the National Cancer Institute Surveillance, Epidemiology and End Results Registry analyzing all cases of appendiceal tumors (carcinoid, appendiceal adenocarcinoma and lymphoma) in children in the United States between 1972 and 2011. Extensive surgery involving a RHC and lymph node sampling based on the presence of an aggressive tumor type, greater disease severity and larger tumor size has not been shown to increase patient survival, and might instead be associated with a greater operative risk [8]. In addition, Wu et al. analyzed 45 appendiceal NET patients over a 20-year period and postulate that postoperative somatostatin scans and serum biomarkers do not seem to be useful and inclined to a less aggressive approach regarding extended surgery beyond appendectomy due to the excellent prognosis [17] (Table 2). Taking into consideration that in our cohort a total of 8 patients with carcinoid tumors underwent RHC with the vast majority treated by adult surgeon, we speculate that this might reflect the discrepancy between the approach towards RHC and recommendations by different national and international societies. Nevertheless, recently published papers generally recommended a less aggressive surgical approach (Table 2). Due to lack of data, especially on the size of the carcinoids, the indications for RHC in these cases cannot be discussed.

Although appendiceal carcinoids are generally found following surgical resection, there is growing evidence of successful conservative management of uncomplicated appendicitis with the risk of leaving a malignancy untreated. If children with appendiceal carcinoids might then be missed by the initial diagnostic evaluation and diagnosed at a later stage leading to a more extensive resection than appendectomy alone, is still unknown and often considered to be negligible due to the low risk of metastasis [23,24]. However, Korsch et al. have recently published two cases of a 13-year old boy and 17-year-old girl with a carcinoid tumor (neuroendocrine tumor, NET) of the appendix, in whom initial conservative therapy of an otherwise uncomplicated acute appendicitis in one case and complicated perforated appendicitis with abscess in the other led to a delay in detection and treatment of appendiceal NET. The authors strongly advise to inform patients and parents about the potential risk of belated diagnosis of this rare malignancy when conservative treatment is chosen. In addition, they recommend a close follow-up by ultrasound for patients successfully conservatively treated and emphasize an interval appendectomy in presence of any conspicuous finding [25].

**Table 2:** Recommendations for additional surgery and work-up/follow-up modalities in children and adolescents from latest publications (sorted by year of publication, adapted from [1]).

Publication	Year	No. of Patients	Recommendations for Additional Surgery	Recommendations for Follow-Up/Work-Up
Boxberger et al. [22] (GPOH-MET trial)	2013	237	Tumor > 1.5 cm: RHC Tumor ≤ 1.5 cm + incomplete resection: ileocecal pole resection and strict lymph node sampling	every 3 months for the first year twice a year for the second year once a year from 3rd to 10th year (abdominal US, CgA,5-HIAA)
Kulkarni and Sergi [26]	2013	7	Tumor > 2 cm with mesoappendix involved or residual tumor: RHC Tumor < 2 cm: appendectomy	Tumor < 2 cm, no metastasis: clinical and imaging follow-up may be adequate, no need of neuroendocrine markers
Kim et al. [5]	2014	13	-	Tumor < 2 cm, no invasion of mesoappendix, complete resection: no follow-up Tumor > 2 cm, invasion of mesoappendix, incomplete resection: 6 monthly 5-HIAA, CgA for 2 years and then yearly for 2 years. MRI/CT imaging at 6 months, followed by yearly for 5 years.
Virgone et al. [27]	2014	113	If Tumor < 2 cm, octreotide scintigraphy positive and 5-HIAA positive If Tumor > 2 cm, suspicious lesions on octreotide scintigraphy, 5-HIAA, US, MRI/CT If Incomplete resection (+ additional diagnostic work-up: octreotide scintigraphy, 5-HIAA, MRI/CT)	If Tumor < 2 cm, octreotide scintigraphy negative/5-HIAA positive or negative If Tumor > 2 cm, no lesions on octreotide scintigraphy, 5-HIAA, US, MRI/CT
Henderson et al. [11]	2014	27	Incomplete excision: RHC	-
de Lambert et al. [6]	2016	114	Not recommended	Not recommended
Wu et al. [7]	2016	45	RHC not recommended “less aggressive approach”	Serum biomarkers and octreotide scintigraphy not recommended
Sommer et al. [1]	2018	40	Tumor ≤ 2 cm, incomplete resection, US, MRI/CT, CgA, 5-HIAA: ileocecal resection and local lymph node sampling (if positive: RHC) Tumor > 2 cm, complete resection, US, MRI/CT, CgA, 5-HIAA positive: RHC Tumor > 2 cm, complete resection, US, MRI/CT, CgA, 5-HIAA negative: ileocecal resection and local lymph node sampling (if positive: RHC) Tumor > 2 cm, incomplete resection: RHC	After RHC: US, CgA, 5-HIAA at 3, 6 months and 1, 2, 4, 6, 8, 10 year(s) after diagnosis If extension to mesoappendix or G2: clinical examination 1, 2, 4, 6, 8, 10 year(s) after diagnosis If ileocecal resection and local lymph node sampling negative: clinical examination 1, 2, 4, 6, 8, 10 year(s) after diagnosis
Yalcin et al. [18]	2022	33	Tumor ≤ 2 cm: appendectomy Incomplete excision: more extensive surgery	-

### 4.6. Limitations

One of the main limitations of this study is the lack of detailed information on histopathological reports, including the size of the individual carcinoid tumors and subsequently the potential indication for RHC. There is the possibility of a selection bias, as only those patients could be analyzed, who were continuously members of the health insurance during the study period. Additional implicit limitations of using anonymized insurance based data embrace missing preoperative data such as ultrasound findings, lack of preoperative information about symptoms and the paucity on complications post discharge (e.g., outpatient treatment for wound infections) [16]. Finally, the observational period of 180 days might be insufficient to draw any conclusions regarding long term outcome and survival, especially for the one patient with metastatic disease. Authors should discuss the results and how they can be interpreted from the perspective of previous studies and of the working hypotheses. The findings and their implications should be discussed in the broadest context possible. Future research directions may also be highlighted.

## 5. Conclusions

This observational study included patients from different surgical centers and presents by far the largest nationwide cohort analyzed in a three- and five-year period, aiming to add further data to the existing literature. The prevalence of appendiceal carcinoids among children with appendicitis in the present study was very low and the majority of patients were treated with appendectomy only. Although established protocols and international guidelines are available, the management of neuroendocrine tumors in children is still controversial and variable, especially indications for extended surgery and follow-up procedures. Therefore, this study emphasizes the urgent need for longer follow-up of children affected by this malignancy up to adulthood, which will help to evaluate on guidelines to pursue a limited surgical approach and postoperative work-up in this particular age group.

## Figures and Tables

**Table 1:** Patient characteristics.

	2010–2012/2016–2020
**Total number of patients with appendectomy**	40.499
**Primary hemicolectomy** (n (%))	2 (5)
**Carcinoid** (n (%))	44 (0.11)
**Sex** (n (%)) *	
Male	12 (29)
Female	29 (71)
n/a	3 (7)
**Appendectomy**	
Mean age (years (±SD))	14.7 (±2.6)
Laparoscopic approach (n (%))	40 (91)
**Admitting Specialty** (n (%)) *	
General Surgery	31 (76)
Pediatrics	5 (12)
Pediatric Surgery	4 (10)
Gynecology	1 (2)
n/a	3 (18)
**Preoperative CT** (n (%))	3 (7)
**Mean length of stay** (days (±SD))	4.7 ± 3.0
**Further admissions (within first 180 days following appendectomy),** (n (%))	
Secondary hemicolectomy	6 (14)
CT	1 (2)
MRI	4 (9)

* based on n = 41 patients, in n = 3 patients data not stated.

## Data Availability

Not applicable.
